# Homebound versus Bedridden Status among Those with Myalgic Encephalomyelitis/Chronic Fatigue Syndrome

**DOI:** 10.3390/healthcare9020106

**Published:** 2021-01-20

**Authors:** Karl Conroy, Shaun Bhatia, Mohammed Islam, Leonard A. Jason

**Affiliations:** Center for Community Research, DePaul University, Chicago, IL 60614, USA; sbhatia3@depaul.edu (S.B.); mislam9@depaul.edu (M.I.); LJASON@depaul.edu (L.A.J.)

**Keywords:** ME/CFS, chronic fatigue syndrome, myalgic encephalomyelitis, illness severity, homebound, bedridden

## Abstract

Persons living with myalgic encephalomyelitis/chronic fatigue syndrome (ME/CFS) vary widely in terms of the severity of their illness. It is estimated that of those living with ME/CFS in the United States, about 385,000 are homebound. There is a need to know more about different degrees of being homebound within this severely affected group. The current study examined an international sample of 2138 study participants with ME/CFS, of whom 549 were severely affected (operationalized as ‘Homebound’). A subsample of 89 very severely affected participants (operationalized as ‘Homebound-bedridden’) was also examined. The findings showed a significant association between severely and very severely affected participants within the post-exertional malaise (PEM) symptom domain. The implications of these findings are discussed.

## 1. Introduction

Myalgic encephalomyelitis/chronic fatigue syndrome (ME/CFS) is a debilitating chronic illness that affects about 0.42% of the adult population [1]. Persons with this disease experience diverse symptoms, including post-exertional malaise, cognitive impairment, and unrefreshing sleep [2,3,4]. When compared to adults with conditions such as cancer, stroke, schizophrenia, and renal failure, those with ME/CFS have reported a lower quality of life [5]. Research has also found that persons with ME/CFS have a poorer prognosis than those with a variety of other serious medical conditions [6].

Because those with ME/CFS vary significantly in their symptom presentation and functional status, study participants have been classified according to four categories of illness severity: (1) mild; (2) moderate; (3) severe; and (4) very severe [7,8,9,10]. Participants classified as ‘mild’ (Grade 1) can work and complete domestic tasks, but are restricted in leisure activities; participants classified as ‘moderate’ (Grade 2) are less mobile, restricted in daily activities, and have stopped working; participants classified at ‘severe’ (Grade 3) can perform only minimal self-care tasks (e.g., face washing, teeth cleaning) and are homebound; and finally, participants classified as ‘very severe’ (Grade 4) cannot complete daily tasks without assistance and are often bedridden [8]. It is also possible for participants to be classified as not homebound (‘mild’ or ‘moderate’), homebound (‘severe’), and bedridden (‘very severe’).

It has been estimated that between 10–25% of persons with ME/CFS might be homebound as they are severely or very severely affected [11,12]. A recent review article of 21 studies examined findings from severely and very severely affected study participants over the past two decades [13]. Most studies were limited by small samples of participants [14,15,16,17,18]. Out of the 21 studies identified by Strassheim and colleagues [13], only four included samples of more than 70 severely or very severely affected participants [8,19,20,21]. Since the review published by Strassheim and colleagues [13], several other studies exploring illness severity in ME/CFS have been published [22,23,24], but in each investigation, the number of participants classified as severely affected was also relatively small.

Among the studies on ME/CFS severity with larger samples, Cox and Findley [8] administered surveys to 72 inpatients who were severely or very severely affected in order to track their perceived activity level (at six-month intervals) and overall symptom duration (in years). However, the survey did not measure dimensions of symptomatology. Pheby and Faffron [19] surveyed 1104 study participants, of whom 124 were severely affected, for the purpose of associating severe ME/CFS to pre-illness risk factors such as occupation, personality type, and smoking or chemical exposure. Although several risk factors for severe ME/CFS were identified (e.g., being a homemaker, exposure to chemicals in the home), the instruments used to measure personality traits had not been validated for persons with ME/CFS, raising concern among other researchers [21]. Additionally, Friedberg and colleagues [20] enrolled 137 severely affected participants in a clinical trial to access the efficacy of fatigue self-management (as opposed to treatment at a clinic), and suggested that self-management might benefit those who are homebound.

Another study that examined a larger sample [21] compared severely affected participants (*n* = 128) to non-severely affected participants (*n* = 409) using a validated measure of ME/CFS symptomatology [25]. The study found that those who were severely affected (operationalized as homebound) reported significantly higher scores for 35 out of 54 symptoms. Furthermore, the study compared participants’ functional status and found that homebound participants reported higher levels of bodily pain and lower levels of physical and social functioning than non-homebound participants.

Nonetheless, the findings presented by Pendergrast and colleagues [21] did not attempt to determine which symptoms were the most predictive of a participant being homebound. In addition, that study did not differentiate those who were homebound and bedridden from those who were homebound but not bedridden. The current study examined predictors of homebound versus not homebound status in participants with ME/CFS, and in a follow up analysis, examined predictors of participants being homebound but not bedridden versus homebound and bedridden.

## 2. Materials and Methods

### 2.1. Participants

The dataset for the current study was aggregated across several international samples as described below.

DePaul sample. An international convenience sample of adults who self-identified as having ME/CFS was collected by investigators at DePaul University. Eligible participants were those at least 18 years of age with a current self-reported and diagnosis of CFS or ME. The sample included 210 participants, of which 83.7% were female. The mean age of participants was 52.1 years (*SD* = 11.2). Most of the participants (74.2%) had completed at least a standard college degree.

BioBank 2016 sample. Collected by the Solve ME/CFS Initiative (https://solvecfs.org), the BioBank sample included participants recruited by physicians who specialized in diagnosing ME/CFS. Following exclusion due to missing data, the final sample consisted of 492 participants. In total, 77.3% of the sample was female with a mean age of 54.6 years (*SD* = 12.0). Seventy percent of participants had completed at least a standard college degree.

Newcastle sample. Participants from the Newcastle sample were those referred to the Newcastle-upon-Tyne Royal Victoria Infirmary clinic for a medical assessment due to a suspected diagnosis of CFS. Following exclusions due to missing data, the final sample consisted of 85 participants. The majority of the participants were female (80.0%) with a mean age of 45.9 years (*SD* = 13.5). Fifty percent of the sample had obtained at least a standard college degree.

Norway 1 sample. Participants from the Norway 1 sample were recruited from southern Norway, and were contacted via healthcare professionals, ME/CFS organizations, and the waiting list for a ME/CFS education program. Participants were required to be at least 18 years of age with a diagnosis of ME/CFS by a physician or medical specialist. Following exclusion due to incomplete data, the final sample consisted of 168 participants. Most participants (87.4%) were female with a mean age of 43.5 years (*SD* = 11.8). Just over half (50.6%) of the sample had completed at least a standard college degree.

Norway 2 sample. Participants from the Norway 2 sample were recruited from two sites: an inpatient medical ward for severely ill patients, and an outpatient clinic at a multidisciplinary ME/CFS center. Eligible participants were those between 18 and 65 years of age, who were able to read and write in Norwegian. Participants underwent a comprehensive medical history and examination conducted by an experienced physician and a psychologist. Following exclusion due to incomplete data, the final sample consisted of 51 participants. Most of the of participants (82.4%) were female with a mean age of 35.8 years (*SD* = 11.9). Approximately 39.2% of the sample had completed at least a standard college degree.

Norway 3 sample. Participants from the Norway 3 sample were recruited while attending a specialist ME/CFS tertiary care center. Eligible participants were those examined by an experienced physician and determined to meet the Canadian Consensus criteria for ME/CFS [3]. Participants were required to be between 18 and 65 years of age. Following exclusion due to incomplete data, the final sample consisted of 167 participants. The majority of the sample (82.0%) was female with a mean age of 38.7 years (*SD* = 11.2). Over half of participants (57.5%) had received at least a standard college degree.

Chronic Illness sample. The Chronic Illness respondents were from a convenience sample of adults living with chronic illnesses, including ME/CFS, collected by investigators at DePaul University [26]. Participants were recruited online using support groups, research forums, and social media platforms. Following the exclusion of participants due to missing data, the final sample consisted of 324 participants with a self-reported diagnosis of ME/CFS. Most of the sample (88.1%) was female with a mean age of 50.1 years (*SD* = 13.5). Most of the participants (70.9%) had completed at least a standard college degree.

Japan sample. Participants from the Japan sample were recruited from the ME Japan Association (https://mecfsjapan.com) and affiliated physician clinics specializing in ME/CFS. In total, 111 were included in the present study following exclusionary procedures due to incomplete data. Much of the sample (79.1%) were female with a mean age of 46.4 years (*SD* = 13.3). A little over half of the sample (52.7%) had completed at least a standard college degree.

Spain sample. Participants from the Spain sample were recruited from a tertiary referral center in Barcelona, Spain by a specialist physician with experience diagnosing ME/CFS. Eligible participants were required to be at least 18 years of age and meet the 1994 Fukuda case definition for CFS [2]. In total, 182 participants were included in the present study following exclusionary procedures due to incomplete data. Most of the sample (85.7%) was female with a mean age of 50.4 years (*SD* = 8.7), and 14.8% of participants had completed a least a standard college degree.

Amsterdam sample. Participants from the Amsterdam sample were selected from an outpatient clinic in the Netherlands (the CFS Medical Center in Amsterdam). Following exclusion due to incomplete data, the final sample consisted of 348 participants, all with physician report of ME/CFS diagnosis. Much of the sample (77.9%) was female with a mean age of 37.1 years (*SD* = 11.5). Under half of the participants (41.4%) had obtained at least a standard college degree.

### 2.2. Measures

The DePaul Symptom Questionnaire. Participants across all datasets completed the DePaul Symptom Questionnaire [25], a 54-item self-report measure of ME/CFS symptomatology. Participants were asked to rate the frequency of each symptom over the past six months on a five-point Likert scale with 0 = none of the time, 1 = a little of the time, 2 = about half the time, 3 = most of the time, and 4 = all of the time. Likewise, participants were asked to rate the severity of each symptom over the past six months on a similar scale with 0 = symptom not present, 1 = mild, 2 = moderate, 3 = severe, and 4 = very severe. All frequency and severity scores were standardized to a 100-point scale. Furthermore, the frequency and severity scores for each symptom were averaged to create a composite score, where higher scores indicated worse symptoms. These item composite scores were averaged, resulting in eight standardized symptom domain scores: (1) sleep dysfunction; (2) post-exertional malaise (PEM); (3) neurocognitive dysfunction; (4) immune dysfunction; (5) neuroendocrine dysfunction; (6) pain; (7) gastro-intestinal distress; and (8) orthostatic intolerance [25].

The DSQ-1 has shown good test-retest reliability among persons with ME/CFS and controls [27] and yielded valid, clinically useful results [28,29]. The DSQ-1 is available in the shared library of Research Electronic Data Capture (REDCap) [30,31] hosted at DePaul University. The full questionnaire can be viewed here: https://redcap.is.depaul.edu/surveys/?s=tRxytSPVVw.

Medical Outcomes Study 36-Item Short-Form Health Survey (SF-36 or RAND Questionnaire). Participants also completed the RAND 36-Item Short-Form Health Survey (SF-36) [32], a self-report measure assessing the impact of health outcomes on physical and mental functioning across eight domains: (1) physical functioning; (2) bodily pain; (3) role physical (limitations due to physical health problems); (4) role emotional (role limitations due to personal or emotional problems); (5) mental health; (6) social functioning; (7) vitality; and (8) general health. All domains are measured on 100-point scales, where higher scores indicate better health functioning.

The SF-36 has produced short- and long-term results that are psychometrically stable [33], has demonstrated strong internal consistency and good discriminant validity [34], and has shown utility across multiple illness groups [35], including fatiguing illnesses such as ME/CFS [36].

### 2.3. Illness Severity Status 

Homebound versus Not Homebound. The DSQ-1 includes an item that asks participants to describe their fatigue/energy related illness over the past six months. Those who responded affirmatively to one of the following items were classified as ‘Homebound:’ “I am not able to work or do anything, and I am bedridden”; “I can walk around the house, but I cannot do light housework.” Participants who responded affirmatively to any of the remaining items were classified as ‘Not homebound’: “I can do light housework, but I cannot work part-time”; “I can only work part-time at work or on some family responsibilities”; “I can work full-time, but I have no energy left for anything else”; and “I can work full-time and finish some family responsibilities, but I have no energy left for anything else” [25]. The group classified as ‘Homebound’ constituted 25.7% of the total sample (549 out of 2138). Although it is possible that some who indicated that they can do light housework but cannot work part-time are actually homebound, we decided to conservatively classify them as not homebound as it is at least conceivable that some within this group were able to engage in some activities outside their houses.

Bedridden versus Not Bedridden. Among those who were classified as ‘Homebound,’ we created two subcategories: ‘Homebound-bedridden’ and ‘Homebound-not bedridden’. Participants who selected “I am not able to work or do anything, and I am bedridden” were classified as ‘Homebound-bedridden,’ whereas participants who selected “I can walk around the house, but I cannot do light housework” were classified as ‘Homebound-not bedridden’. The group classified as ‘Homebound-bedridden’ comprised 16.2% of the original ‘Homebound’ group (89 out of 549) and the ‘Homebound-not bedridden’ group represented the remaining 83.8% (460 out of 549).

### 2.4. Statistical Procedure

Demographics. Chi-squared tests were conducted to determine if significant differences were present for demographic characteristics (gender, educational status, and marital status) across severity classifications. Independent samples *t*-tests were conducted to determine if significant differences in age were present. Variables which indicated the presence of demographic heterogeneity were included in base logistic regression models. These models were developed for two severity analyses (‘Homebound’ compared to ‘Not homebound’ and ‘Homebound-bedridden’ compared to ‘Homebound-not bedridden’). 

Binary logistical regression. The criterion variable for our first analysis was ‘Homebound’ status compared to ‘Not homebound’ status, whereas the criterion variable for our second analysis was ‘Homebound-bedridden’ status compared to ‘Homebound-not bedridden’ status. Using a top-down analytic approach, we performed binary logistical regressions on the DSQ-1 and SF-36 domains, where each DSQ-1 domain represented a linear combination of individual symptom items [25]. To reduce chance findings with so many potential comparisons, we initially focused on DSQ-1 domains, and if significance was found within a domain, symptom items within those domains were examined in a later step. This multi-step process for variable selection [37] was chosen to facilitate an efficient analysis of the DSQ-1’s extensive symptom inventory (54 items). 

The demographic base models were used when testing the DSQ-1 and SF-36 domains, with each domain being tested individually (Step 1). Domains that were observed to be statistically significant in predicting severity status were entered into a forward stepwise selection procedure using likelihood ratio tests (Step 2). 

If a statistically significant domain was identified in the forward stepwise procedure, we tested the domain’s component symptom scores individually with the demographic base model and all statistically significant SF-36 domains (Step 3). In the development of the final models, those symptoms that were found to be statistically significant were entered into another forward stepwise selection procedure based on likelihood ratio tests (Step 4). IBM SPSS Statistics version 25 was used for all analyses [38]. 

In summary, rather than utilizing a statistical approach similar to Pendargrast and colleagues [21] to detect mean differences in specific symptoms in our ME/CFS severity groups, our iterative regression process specified two a priori group comparisons that were of interest (i.e., ‘Homebound’ versus ‘Not homebound’ and ‘Homebound-bedridden’ versus ‘Homebound-not bedridden’). In preliminary work, we did find significant differences in symptoms and functionality between the three groups of participants, but our intent in the current study was to investigate two sets of comparisons among the illness severity groups. 

## 3. Results

### 3.1. Demographics

Table 1 shows the demographic characteristics of the ‘Homebound’ versus ‘Not homebound’ groups. Statistical differences were observed in gender [χ^2^ (1, 2, 112) = 13.07, *p* < 0.001] and educational status [χ^2^ (1, 2, 106) = 15.71, *p* < 0.001]. The ‘Homebound’ group compared to the ‘Not homebound’ group had a smaller percentage of male participants (13.0% compared to 19.9%) and a smaller percentage of participants who had completed at least a standard college degree (48.9% compared to 58.7%). Based on these findings, subsequent regression analysis of the ‘Homebound’ group compared to the ‘Not homebound’ group was adjusted for gender and educational status.

Additionally, Table 1 shows the demographic characteristics for two sub-divisions of the ‘Homebound’ group: ‘Homebound-not bedridden’ and ‘Homebound-bedridden’. Significant differences were observed in age [*t*(526) = −6.79, *p* < 0.001] and marital status [χ^2^ (1, 539) = 4.63, *p* = 0.031]. The ‘Homebound-bedridden’ group was significantly younger than the ‘Homebound-not bedridden’ group (mean ages were 37.5 and 48.3, respectively) and fewer participants were married (40.9% compared to 53.4%). Subsequent regression analysis of the ‘Homebound-bedridden’ group compared to the ‘Homebound-not bedridden’ group was adjusted for age and marital status.

### 3.2. Homebound Status 

Table 2 (Step 1) shows the regression results for each DSQ-1 and SF-36 domain, tested individually and adjusted for gender and educational status. Every SF-36 and DSQ-1 domain was found to be a statistically significant predictor of ‘Homebound’ status. Table 2 (Step 2) shows the results of a forward stepwise selection procedure of statistically significant predictors from Step 1. Regarding the DSQ-1 domains, more severe scores in the PEM domain increased the odds of a participant being ‘Homebound’ [odds ratio (OR) = 1.034, 95% CI, (1.025, 1.044)]; no other DSQ-1 domains were found to be statistically significant. Regarding the SF-36 domains, higher levels of physical functioning and social functioning decreased the odds of a participant being ‘Homebound’ [OR = 0.957, 95% CI, (0.950, 0.965); OR = 0.980, 95% CI, (0.974, 0.987)]. 

Table 2 (Step 3) shows regression results for every symptom that constitutes the PEM domain, tested individually and adjusted for demographics, physical functioning, and social functioning. “Dead, heavy feeling after starting to exercise,” “next day soreness or fatigue after non-strenuous, everyday activity,” “mentally tired after the slightest effort,” “minimum exercise makes you physically tired,” and “physically drained or sick after mild activity” were found to be statistically significant predictors of ‘Homebound’ status; “muscle weakness” was not a significant predictor. Table 2 (Step 4) shows the results of a second forward stepwise selection of statistically significant predictors from Step 3, adjusted for gender, educational status, physical functioning, and social functioning. Regarding the symptoms, more severe scores for “next day soreness or fatigue after non-strenuous, everyday activity” and “physically drained or sick after mild activity” both increased the odds of a participant being ‘Homebound’ [OR = 1.016, 95% CI, (1.008, 1.025); OR = 1.024, 95% CI, (1.015, 1.032)]; no other symptoms were statistically significant. 

### 3.3. Bedridden Status

Table 2 (Step 1) shows the regression results for each DSQ-1 and SF-36 domain, tested individually and adjusted for age and marital status. The PEM and neurocognitive dysfunction domains were the only statistically significant predictors of a participant being ‘Homebound-bedridden’. Table 2 (Step 2) shows the results of a forward stepwise selection operation of statistically significant domain scores from Step 1 (PEM and neurocognitive dysfunction), adjusted for age and marital status. The only statistically significant domain was PEM, where more severe scores decreased the odds of a participant being ‘Homebound-bedridden’ (compared to ‘Homebound-not bedridden’) [OR = 0.974, 95% CI, (0.959, 0.989)]. 

Table 2 (Step 3) shows regression results for every symptom that constitutes the PEM domain, tested individually and adjusted for age and marital status. “Dead, heavy feeling after starting to exercise,” “next day soreness or fatigue after non-strenuous, everyday activity,” “minimum exercise makes you physically tired,” and “physically drained or sick after mild activity” were all significant predictors of a participant being ‘Homebound-bedridden’; “mentally tired after the slightest effort” and “muscle weakness” were not significant. Table 2 (Step 4) shows the results of another forward stepwise selection operation of statistically significant predictors from Step 3, adjusted for age and marital status. Regarding the symptoms, more severe scores for “minimum exercise makes you physically tired” decreased the odds of a participant being ‘Homebound-bedridden’ (compared to ‘Homebound-not bedridden’) [OR = 0.974, 95% CI, (0.961, 0.988)]; no other symptoms were statistically significant. 

## 4. Discussion

The findings of the current study indicated that PEM, social functioning, and physical functioning were significant predictors of a participant with ME/CFS being ‘Homebound’ (compared to ‘Not homebound’). Among symptom items in the DSQ-1 PEM domain, “next day soreness or fatigue after non-strenuous, everyday activity” and “physically drained or sick after mild activity” were the strongest predictors of ‘Homebound’ status. These predictive results were consistent with the mean comparisons reported by Pendergrast and colleagues [21]. Moreover, the unique aspect of our study was subdividing the ‘Homebound’ group into two subgroups: ‘Homebound-bedridden’ and ‘Homebound-not bedridden. We found that higher symptom scores in the PEM domain decreased the odds of a participant being ‘Homebound-bedridden’ (versus ‘Homebound-not bedridden’). Among the PEM symptom items, “minimum exercise makes you physically tired” significantly decreased the odds of a participant being ‘Homebound-bedridden.

Although several studies have mentioned the need for research that differentiates those with ME/CFS at varying levels of illness severity [3,8,39], existing research has focused on differences between participants who are severely and moderately affected [16,17,18,20,21,22,23,24] but not severely and very severely affected. While the illness severity gradation (i.e., mild, moderate, severe, very severe) proposed by Cox and Findley [8] describes differences between those who are severely affected (homebound) and those who are very severely affected (bedridden) in terms of symptom presentation (e.g., those who are bedridden might be sensitive to noise and light), their distinctions lacked an empirical foundation.

According to our findings, participants who were ‘Homebound’ (compared to participants who were ‘Not homebound’) were at increased odds of being less physically and socially functional, as well as exhibiting PEM symptomology, where “next day soreness or fatigue after non-strenuous, everyday activity” and “physically drained or sick after mild activity” had the strongest effect among the PEM items tested at Step 4. Inversely, participants who were ‘Homebound-bedridden (compared to participants who were ‘Homebound-not bedridden’) were at decreased odds of exhibiting PEM symptomology, where “minimum exercise makes you physically tired” had the strongest effect at Step 4. These findings can be explained by the fact that participants who are bedridden (very severely affected) have fewer opportunities to engage in activities. For example, if a severely affected ‘Homebound-not bedridden’ participant with ME/CFS is expending significant amounts of their limited energy around their household, they may risk triggering more PEM symptoms than very severely affected ‘Homebound-bedridden’ participants who are less active. Indeed, when we compared the ‘Homebound-bedridden’ group to the ‘Homebound-not bedridden’ group at Step 3 using the component symptoms within the PEM domain, we found that symptoms involving activity such as “dead, heavy feeling after starting to exercise,” “next day soreness or fatigue after non-strenuous, everyday activity,” “minimum exercise makes you physically tired,” and “physically drained or sick after mild activity” were statistically significant, whereas symptoms that did not explicitly involve activity, such as “mentally tired after the slightest effort” and “muscle weakness,” were not statistically significant, which could mean that PEM triggered by mental exertion is experienced more evenly between the two groups. These findings suggest that treatment programs targeting persons who are homebound with ME/CFS should account for the heterogeneity of this population (i.e., bedridden and not bedridden). The symptomological differences identified in our study highlight the need for treatment programs that are tailored for two subgroups.

Of interest, the proportion of participants we classified as severely affected (25.7%, 549/2138) matched estimates offered by ME/CFS advocacy groups [11,12]. A recent study [40] estimated that 1.5 million persons suffer from ME/CFS in the United States. If 25.7% of those with ME/CFS are not able to leave their homes, there may be as many as 385,000 persons in the US who are homebound due to ME/CFS. Furthermore, our study found that 16.2% (89/549) of those who were homebound with ME/CFS were also bedridden, which equates to roughly 62,000 persons in the US. These estimates indicate a serious public health problem, as many who are homebound or bedridden due to ME/CFS may lack access to the healthcare system. Providing this group with adequate services will require attention and resources at many levels (e.g., research, treatment, and policy-making). We maintain that a crucial first step is to focus research on those who are severely and very severely affected, which will require methods that are sensitive to the needs of this population [39].

The current study had two limitations. First, the total sample (*n* = 2138) was aggregated from multiple sources, so there were inconsistencies in terms of participant recruitment and assessment. While a number of sources recruited participants who had complete a full medical review (e.g., Norway 1–3 samples, Spain sample), others allowed participants to self-report their medical diagnosis (e.g., DePaul sample, Chronic Illness sample). Second, our study used a variety of case ascertainment methods from recruitment using the internet to tertiary care settings, but such methods might have also increased the generalizability of the findings.

Our study found that participants who reported worse symptoms in the PEM domain [25] and less physical and social functioning [32] were at increased odds of being ‘Homebound’ (compared to ‘Not homebound’). Among participants who were classified as ‘Homebound,’ those who reported worse symptoms in the PEM domain were at decreased odds of being ‘Homebound-bedridden’ (compared to ‘Homebound-not bedridden’). We hypothesized that for participants who are ‘Homebound,’ those who are ‘Homebound-bedridden’ may experience less PEM symptomology because they are expending less energy. Based on the proportion of participants who were ‘Homebound’ in our study, we estimate that as many as 385,000 persons with ME/CFS are homebound in the United States. There is a pressing need to find ways of providing services to this under-resourced group.

## Figures and Tables

**Table 1:** Demographic characteristics.

	Total Sample Size (*n* = 2138)			Homebound (*n* = 549)		
Characteristics	Homebound	Not Homebound	*p*	Bedridden	Not Bedridden	*p*
	(*n* = 549)	(*n* = 1589)		(*n* = 89)	(*n* = 460)	
	*M* (*SD*)	*M* (*SD*)		*M* (*SD*)	*M* (*SD*)	
Age	46.6 (14.0)	47.2 (13.5)	0.393	37.5 (12.1)	48.3 (13.7)	<0.001
	% (*n)*	% (*n)*		% (*n)*	% (*n)*	
Gender			<0.001			0.111
Male	13.0 (70)	19.9 (313)		18.2 (16)	11.9 (54)	
Female	87.0 (470)	80.1 (1259)		81.8 (72)	88.1 (398)	
Education			<0.001			0.811
At least a standard college degree	48.9 (265)	58.7 (918)		47.7 (42)	49.1 (223)	
Less than a standard college degree	51.1 (277)	41.3 (646)		52.3 (46)	50.9 (231)	
Marital			0.071			0.031
Married	51.4 (277)	55.9 (875)		40.9 (36)	53.4 (241)	
Not married	48.6 (262)	44.1 (691)		59.1 (52)	46.6 (210)	

**Table 2:** Binary logistic regressions predicting ‘Homebound’ status compared to ‘Not homebound’ status and ‘Homebound-bedridden status compared to ‘Homebound-not bedridden’ status.

	Homebound ^a^	Bedridden ^b^
Iteration	e ^b^ (95% CI)	e ^b^ (95% CI)
Step 1		
DSQ-1 domain		
Sleep	1.028 (1.022, 1.033)	1.005 (0.992, 1.017)
PEM	1.071 (1.063, 1.080)	0.974 (0.959, 0.989)
Neurocognitive	1.028 (1.022, 1.033)	0.985 (0.975, 0.997)
Immune	1.028 (1.021, 1.033)	0.998 (0.986, 1.010)
Neuroendocrine	1.015 (1.010, 1.019)	0.989 (0.978, 1.000)
Pain	1.018 (1.014, 1.022)	0.993 (0.984, 1.002)
Gastro-intestinal	1.012 (1.009, 1.016)	0.998 (0.989, 1.007)
Orthostatic	1.033 (1.027, 1.038)	0.993 (0.982, 1.005)
SF-36 domain		
Physical functioning	0.935 (0.929, 0.942)	1.000 (0.987, 1.014)
Role physical	0.965 (0.952, 0.978)	0.986 (0.951, 1.022)
Bodily pain	0.970 (0.964, 0.975)	1.001 (0.991, 1.012)
General health	0.967 (0.960, 0.974)	0.999 (0.982, 1.016)
Vitality	0.964 (0.957, 0.972)	1.006 (0.988, 1.026)
Social functioning	0.956 (0.950, 0.962)	1.004 (0.992, 1.017)
Role emotional	0.996 (0.994, 0.998)	1.003 (0.997, 1.008)
Mental health	0.988 (0.983, 0.993)	0.996 (0.985, 1.007)
Step 2		
Domain		
Age	-	0.937 (0.918, 0.956)
Marital status	-	0.802 (0.480, 1.339)
Gender	0.778 (0.612, 0.988)	-
Grade	0.841 (0.597, 1.186)	-
PEM	1.034 (1.025, 1.044)	0.974 (0.959, 0.989)
Physical functioning	0.957 (0.950, 0.965)	-
Social functioning	0.980 (0.974, 0.987)	-
Step 3		
DSQ-1 Symptom		
Heavy feeling	1.007 (1.002, 1.012)	0.988 (0.979, 0.997)
Soreness after activities	1.029 (1.021, 1.036)	0.979 (0.966, 0.992)
Mentally tired	1.013 (1.007, 1.018)	0.989 (0.978, 1.000)
Minimum exercise	1.027 (1.018, 1.034)	0.975 (0.962, 0.988)
Feeling drained	1.032 (1.024, 1.040)	0.977 (0.963, 0.989)
Muscle weakness	1.003 (0.998, 1.008)	0.997 (0.988, 1.006)
Step 4		
Variable		
Age	-	0.936 (0.917, 0.955)
Marital status	-	0.767 (0.453, 1.299)
Grade	0.813 (0.637, 1.038)	-
Gender	0.880 (0.620, 1.250)	-
Minimum exercise	-	0.974 (0.961, 0.988)
Soreness after activities	1.016 (1.008, 1.025)	-
Feeling drained	1.024 (1.015, 1.032)	-
Physical functioning	0.955 (0.948, 0.963)	-
Social functioning	0.985 (0.978, 0.992)	-

^a^ ‘Homebound’ compared to ‘Not homebound, ^b^ ‘Homebound-bedridden’ compared to ‘Homebound-not bedridden’.

## Data Availability

The data presented in this study are available on request from the corresponding author.
